# Comment on Cesak et al. Carnosine and Beta-Alanine Supplementation in Human Medicine: Narrative Review and Critical Assessment. *Nutrients* **2023**, *15*, 1770

**DOI:** 10.3390/nu16152522

**Published:** 2024-08-02

**Authors:** Robert Child

**Affiliations:** 1 School of Chemical Engineering, University of Birmingham, Birmingham B15 2TT, UK; robchild@alimentarius.eu; 2 Alimentarius Co., Ltd., Brighton BN3 8LE, UK

The paper by Cesak et al. 2023 [1], titled ‘Carnosine and Beta-Alanine Supplementa-tion in Human Medicine: Narrative Review and Critical Assessment’, thoroughly re-views the importance of carnosine in human health. However, I was surprised to read that asparagus, green peas and white mushrooms were considered dietary sources of carnosine. To substantiate this claim, Cesak et al. cite the paper of Jones et al. 2011 [2]; however, these authors only reported the carnosine concentration of meat, fish and shellfish. In animals, carnosine’s imidazole ring has a well-recognised role in intracellular acid buffering [3,4]. In contrast, plants and fungi use very different intracellular systems to counter the challenges of low pH [5,6]. Even though the imidazole group of the amino acid histidine facilitates intracellular pH regulation in plants [5,7], in 2013 it was reported that histidine dipetides (which include carnosine) had never been detected in plants, fungi or other eukaryotes [8]. In light of this, could Cesak et al. provide a reference demonstrating the presence of carnosine in asparagus, green peas and white mushrooms?
